# Rhythm Control in Patients With Recently Diagnosed Atrial Fibrillation: Findings From the GLORIA‐AF Registry Phase III

**DOI:** 10.1161/JAHA.125.044293

**Published:** 2026-03-04

**Authors:** Bernadette Corica, Giulio Francesco Romiti, Marco Proietti, Davide Antonio Mei, Giuseppe Boriani, Brian Olshansky, Menno V. Huisman, Gregory Y.H. Lip, the GLORIA‐AF Investigators

**Affiliations:** ^1^ Liverpool Centre for Cardiovascular Science at University of Liverpool Liverpool John Moores University and Liverpool Heart and Chest Hospital Liverpool UK; ^2^ Cardiology Division, Department of Biomedical, Metabolic and Neural Sciences University of Modena and Reggio Emilia, Policlinico di Modena Modena Italy; ^3^ Department of Wellbeing, Health and Environmental Sustainability Sapienza—University of Rome Rome Italy; ^4^ Department of Clinical Sciences and Community Health University of Milan Milan Italy; ^5^ Division of Cardiogeriatric Subacute Care IRCCS Istituti Clinici Scientifici Maugeri Milan Italy; ^6^ Division of Cardiology, Department of Internal Medicine University of Iowa Hospitals and Clinics Iowa City IA USA; ^7^ Department of Thrombosis and Hemostasis Leiden University Medical Center Leiden the Netherlands; ^8^ Department of Clinical Medicine, Danish Center for Health Services Research Aalborg University Aalborg Denmark; ^9^ Medical University of Bialystok Bialystok Poland

**Keywords:** atrial fibrillation, death, outcomes, rhythm control, treatment strategies, Atrial Fibrillation

## Abstract

**Background:**

Early rhythm control has been proposed to improve outcomes in patients with atrial fibrillation (AF), but data on its effectiveness in real‐world cohorts remain limited. We aimed to evaluate the effectiveness of rhythm control in patients with recently diagnosed AF.

**Methods:**

We included patients with recently diagnosed AF enrolled in the GLORIA‐AF (Global Registry on Long‐Term Oral Antithrombotic Treatment in Patients with Atrial Fibrillation) registry phase III. We analyzed rhythm control at baseline, defined as treatment with an antiarrhythmic drug, or having received AF ablation or cardioversion; patients who did not receive any of these treatments were assigned to the “no rhythm control” group. We analyzed factors associated with rhythm control, treatment with an oral anticoagulant, and risk of major outcomes using multivariable regression analyses. The primary outcome for this analysis was the composite of all‐cause death and major adverse cardiovascular events.

**Results:**

Of 21 051 patients with AF included in this analysis (mean age, 70.2±10.3 years, 45% women), 6932 (32.9%) received rhythm control. Older age, more sustained forms of AF, and history of thromboembolism were associated with no rhythm control at baseline; conversely, oral anticoagulants were more likely used in patients receiving rhythm control (odds ratio, 1.36 [95% CI, 1.25–1.48]). During 3‐year follow‐up, rhythm control was associated with lower hazard of the primary composite outcome (hazard ratio, 0.88 [95% CI, 0.80–0.96]). Similar results were observed for other secondary outcomes, including all‐cause death, thromboembolism, and major bleeding.

**Conclusions:**

In this real‐world cohort of patients with AF, rhythm control was used in 1 of 3 patients, and was associated with higher use of oral anticoagulants and better outcomes.

## Graphical abstract

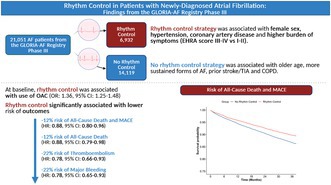

**Nonstandard Abbreviations and Acronyms**
AFFIRM: Atrial Fibrillation Follow‐Up Investigation of Rhythm Management
EAST‐AFNET 4: Early Treatment of Atrial Fibrillation for Stroke Prevention Trial 4
ERC: early rhythm control
GLORIA‐AF: Global Registry on Long‐Term Oral Antithrombotic Treatment in Patients With Atrial Fibrillation
MACE: major adverse cardiovascular event
OAC: oral anticoagulant
RAFAS: Risk and Benefits of Urgent Rhythm Control of Atrial Fibrillation in Patients With Acute Stroke

**Clinical Perspective**

**What Is New?**

- In a real‐world prospective cohort of patients with a recent diagnosis of atrial fibrillation, a rhythm control strategy was used in 32.9% of patients, and was associated with higher use of oral anticoagulants and better outcomes.

**What Are the Clinical Implications?**

- Our findings support the need to personalize management of atrial fibrillation and to consider offering a rhythm control strategy in patients with recently diagnosed atrial fibrillation.

Guideline‐recommended management of atrial fibrillation (AF) is based on a holistic approach to provide comprehensive management of all relevant aspects of AF, including thromboembolic risk, symptoms, and management of comorbidities.^1^, ^2^, ^3^, ^4^ While slightly different versions of these care strategies have been proposed by different guidelines,^1^, ^2^, ^3^, ^4^ all pathways include a specific focus on optimizing symptom control. In the “Atrial Fibrillation Better Care” pathway, proposed in 2017 to streamline a holistic approach to AF care,^5^ better symptom management is implemented through rational, patient‐centered, and symptom‐directed decisions on rhythm versus rate control.^5^

From a prognostic point of view, rate control has been historically deemed noninferior to rhythm control in patients with AF, as found in the AFFIRM (Atrial Fibrillation Follow‐Up Investigation of Rhythm Management) trial^6^; indeed, subsequent studies did not provide evidence of the superiority of a rhythm control approach over rate control.^7^, ^8^ In 2020, the EAST‐AFNET 4 (Early Treatment of Atrial Fibrillation for Stroke Prevention Trial 4) trial demonstrated that in patients with AF diagnosed <12 months before enrollment, an “early rhythm control” (ERC) approach, implemented with antiarrhythmic drugs, electrical cardioversion, or AF ablation, reduced the risk of cardiovascular outcomes compared with a usual care strategy.^9^ These results were subsequently confirmed in specific subgroups of patients with AF, including those with heart failure and high comorbidity burden.^10^, ^11^ Observational studies provided further evidence of the benefits of ERC,^12^, ^13^, ^14^ although some uncertainties were found in selected cohorts.^15^ Nonetheless, variation in the implementation of rhythm control in real‐world practice has been described,^16^ and several characteristics and clinical factors have been associated with the indication for rhythm versus rate control in patients with AF, including age, sex, type of AF, comorbidities, and burden of symptoms.^17^, ^18^ Therefore, more data are needed on the use of rhythm control in real‐world patients with AF.

In this study, we aimed to explore the association of a rhythm control strategy with clinical outcomes in a real‐world cohort of patients with a recent diagnosis of AF. We sought a global perspective, using data from the prospective and multicenter GLORIA‐AF (Global Registry on Long‐Term Oral Antithrombotic Treatment in Patients With Atrial Fibrillation) registry, which enrolled patients with a recent diagnosis of AF across different continents, thus allowing us to investigate the effectiveness of a rhythm control strategy performed early in the natural history of the disease.

## Methods

Data supporting this study from the data contributors, Boehringer Ingelheim, have been made and are available through Vivli, Inc. Access was provided after a proposal was approved by an independent review committee identified for this purpose and after receipt of a signed data‐sharing agreement.

### Study Design

GLORIA‐AF is a multicenter, prospective registry structured in 3 phases that aimed to evaluate the long‐term safety and effectiveness of dabigatran etexilate in patients with AF. Further details of the study design and primary analyses have been previously reported and published.^19^, ^20^, ^21^ For the purpose of this study, we used data from the phase III of the registry, which enrolled patients between January 2014 and December 2016.

### Inclusion/Exclusion Criteria

Detailed inclusion and exclusion criteria have been published previously.^20^ Eligible patients for the GLORIA‐AF registry were adults (aged ≥18 years), with a recent diagnosis of AF (<3 months before baseline visit or <4.5 months in Latin America) and a CHA_2_DS_2_‐VASc score ≥1. All participants provided written informed consent. Patients were excluded if AF was due to a reversible cause, had a mechanical heart valve (or expected valve replacement), received >60 days of treatment with a vitamin K antagonist during their lifetime, had another medical indication for oral anticoagulants (OACs), or had a life expectancy <1 year. The study was conducted following the principles of Good Clinical Practice and the Declaration of Helsinki. Local institutional review boards at each participating site gave ethical approval.

At enrollment, investigators collected data on clinical characteristics, comorbidities, and treatments for each patient, using standardized electronic case report forms. Considering the data recorded by investigators at baseline, we included in the “rhythm control group” all patients receiving either an antiarrhythmic drug (flecainide, propafenone, amiodarone, or dronedarone) or having undergone catheter ablation or electrical cardioversion after confirmation of AF diagnosis. The comparison group, “no rhythm control,” included patients for whom none of these treatments were recorded at baseline. Patients who could not be assigned to either of the 2 groups because of missing data were excluded.

### Follow‐Up and Outcomes

Detailed descriptions of follow‐up and outcomes for the phase III of the GLORIA‐AF registry has been already reported elsewhere.^20^ Briefly, patients enrolled in the phase III of the GLORIA‐AF registry were followed for up to 3 years for the occurrence of major clinical outcomes. For this analysis, we considered the following outcomes: (1) all‐cause death; (2) major adverse cardiovascular events (MACEs; defined as the composite of cardiovascular death, stroke, and myocardial infarction); (3) thromboembolism (defined as the composite of stroke, transient ischemic attack, and other non–central nervous system thromboembolism); and (4) major bleeding (a life‐threatening or fatal bleed, a symptomatic bleed in a critical organ, or a bleed associated with a hemoglobin reduction of ≥20 g/L or leading to a blood transfusion of ≥2 units). For this analysis, we considered the composite of all‐cause death and MACEs as our primary outcome; the other outcomes were also assessed as secondary exploratory outcomes.

### Statistical Analysis

Continuous variables were reported according to mean±SD or median and interquartile range and were compared with appropriate parametric and nonparametric tests. Categorical variables, reported as frequencies and percentages, were compared using the χ^2^ test. We performed multiple‐adjusted logistic regressions to evaluate factors associated with receiving rhythm control at baseline; results were reported as odds ratio (OR) and 95% CI; additionally, we explored the association of age (modeled as a continuous variable using a restricted cubic spline, with 3 knots at default placement) and odds of receiving rhythm control at baseline. Covariates were assessed for multicollinearity using variance inflation factors and excluded accordingly.

Similarly, multiple‐adjusted logistic regression analysis was performed to evaluate the association of rhythm control with use of an OAC at baseline and with use of a non–vitamin K antagonist OAC versus a vitamin K antagonist in those who received an OAC at baseline. These models were adjusted for components of the CHA_2_DS_2_‐VASc score (age class, sex, congestive heart failure, arterial hypertension, diabetes, history of stroke/transient ischemic attack, peripheral artery disease, coronary artery disease), type of AF, body mass index, and history of previous bleeding. Results were reported as OR and 95% CI.

For our primary outcome, we reported Kaplan–Meier curves, and survival distributions were compared using the log‐rank test. Multivariable Cox regression analyses were also performed to evaluate the association of rhythm control with the hazard of the primary and secondary outcomes. Regression models were adjusted for components of the CHA_2_DS_2_‐VASc score, type of AF, body mass index categories, and history of previous bleeding; we additionally included chronic obstructive pulmonary disease, symptoms at baseline (according to European Heart Rhythm Association score, III–IV versus I–II), use of OACs, and geographic region of recruitment as covariates. Results were reported as hazard ratio (HR) and 95% CI. Proportional hazard assumption for the association of rhythm control with outcomes was assessed through visual inspection of scaled Schoenfeld residuals, showing no substantial violations of the proportionality.

We finally performed an exploratory analysis to evaluate if the association of rhythm control with the hazard of the primary outcome differed across key specific subgroups, by evaluating 2‐way interactions with components of the CHA_2_DS_2_‐VASc score, type of AF, body mass index categories, history of previous bleeding, chronic obstructive pulmonary disease, presence of multimorbidity (defined as ≥2 chronic disease among comorbidities included in the CHA_2_DS_2_‐VASc, history of previous bleeding, abnormal kidney function, dementia, history of neoplasia, chronic obstructive pulmonary disease, hepatic disease, obesity and hyperlipidemia, as collected at baseline by investigators), symptoms at baseline, use of an OAC, and geographic region of recruitment. We additionally explored the interaction of rhythm control with age (modeled as a restricted cubic spline with 3 knots at default placement) on the risk of the primary outcome. Fine–Gray subdistributional hazard models were also performed as a sensitivity analysis for MACE, thromboembolism, and major bleeding.

A 2‐sided *P*<0.05 was considered as statistically significant. All analyses were performed using R version 4.3.1 (R Core Team 2020, Vienna, Austria).

## Results

In total, 21 051 patients with AF (age, 70.2±10.3 years; 45% women) with available data were included in this analysis. Of these, 6932 (32.9%) received rhythm control at baseline, and 14 119 (67.1%) were considered in the no rhythm control group. Baseline characteristics according to rhythm versus no rhythm control groups are shown in Table 1; treatments according to these 2 groups are shown in Table S1.

**Table 1:** Baseline Characteristics According to Treatment Groups

Variables	No rhythm control (n=14 119)	Rhythm control (n=6932)	*P* value
Age, y, mean±SD	71.2±10.0	68.1±10.7	<0.001*
Female sex, n (%)	6241/14 119 (44.2)	3222/6932 (46.5)	0.002*
Body mass index, median (IQR)	27.4 (24.3–31.3)	27.5 (24.6–31.3)	0.175
Region, n (%)			<0.001*
North America	3435/14 119 (24.3)	1606/6932 (23.2)	
Europe	6975/14 119 (49.4)	3216/6932 (46.4)	
Asia	2689/14 119 (19.0)	1522/6932 (22.0)	
Other (Latin America)	1020/14 119 (7.2)	588/6932 (8.5)	
AF type, n (%)			<0.001*
Paroxysmal	7513/14 119 (53.2)	4356/6932 (62.8)	
Persistent	4900/14 119 (34.7)	2288/6932 (33.0)	
Permanent	1706/14 119 (12.1)	288/6932 (4.2)	
European Heart Rhythm Association score III–IV, n (%)	3646/14 119 (25.8)	2912/6932 (42.0)	<0.001*
Medical history, n (%)			
Arterial hypertension	10 484/14 093 (74.4)	5230/6919 (75.6)	0.063
Heart failure	2993/13 997 (21.4)	1597/6902 (23.1)	0.004*
Coronary artery disease	2595/13 760 (18.9)	1346/6785 (19.8)	0.098
Diabetes	3338/14 119 (23.6)	1563/6932 (22.5)	0.080
Hyperlipidemia	5604/13 783 (40.7)	2644/6780 (39.0)	0.023
Peripheral artery disease	429/14 011 (3.1)	190/6889 (2.8)	0.240
Previous stroke/transient ischemic attack	2362/14 118 (16.7)	643/6932 (9.3)	<0.001*
Previous bleeding	779/13 848 (5.6)	342/6842 (5.0)	0.066
Abnormal kidney function	266/13 936 (1.9)	122/6882 (1.8)	0.530
Dementia	104/13 956 (0.7)	20/6892 (0.3)	<0.001*
History of cancer	1487/13 914 (10.7)	611/6859 (8.9)	<0.001*
Chronic obstructive pulmonary disease	911/13 960 (6.5)	361/6894 (5.2)	<0.001*
Hepatic disease	187/13 924 (1.3)	122/6884 (1.8)	0.019*
Multimorbidity (≥2 diseases)	6737/14 119 (47.7)	3030/6932 (43.7)	<0.001*
Risk scores, median (IQR)			
CHA_2_DS_2_‐VASc	3 (2–4)	3 (2–4)	<0.001*
HAS‐BLED	1 (1–2)	1 (1–2)	<0.001*

AF indicates atrial fibrillation; and IQR, interquartile range.

* *P* value < 0.05.

Patients in the rhythm control group were younger, more frequently had paroxysmal AF, and were more symptomatic. They also showed a higher prevalence of heart failure, lower prevalence of history of thromboembolism, and lower mean CHA_2_DS_2_‐VASc score (3.0±1.5 versus 3.3±1.5). AF ablation was reported in 5.7% of patients in the “rhythm control” group. Amiodarone (41.7%) was the most common antiarrhythmic drug received in the rhythm control group, followed by flecainide (10.4%) and propafenone (9.4%) (Table S1). Amiodarone was more used in Latin America compared with the other geographic regions, while use of propafenone and AF ablation were most commonly reported in patients recruited in Asia and cardioversion more commonly in Europe and North America (Table S2).

### Factors Associated With Rhythm Control and Association With OAC Use

**Figure 1:**
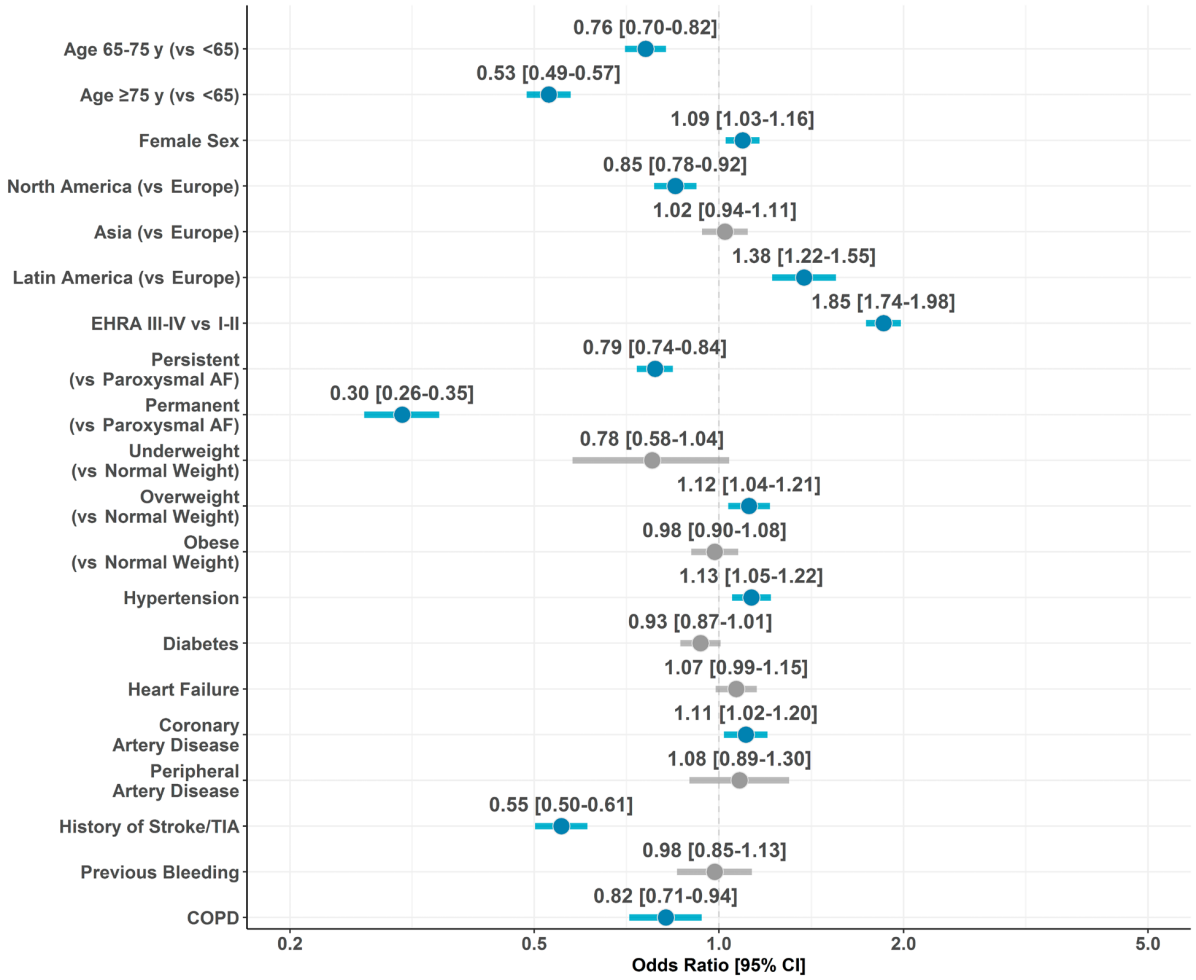
Factors associated with rhythm control at baseline. AF indicates atrial fibrillation; COPD, chronic obstructive pulmonary disease; EHRA, European Heart Rhythm Association; and TIA, transient ischemic attack.

Results of the multivariable logistic regression on rhythm control at baseline are reported in Figure 1 (Hosmer–Lemeshow test, *P*=0.169, C‐statistic=0.658). Older age (OR, 0.76 [95% CI, 0.70–0.82]; and OR, 0.53 [95% CI, 0.49–0.57] for 65–75 versus <65 and ≥75 versus <65 years, respectively) and more sustained forms of AF were associated with lower odds of receiving rhythm control at baseline. Similar results were found for patients recruited in North America (compared with Europe) and for patients with history of stroke/transient ischemic attack (OR, 0.55 [95% CI, 0.50–0.61]) and chronic obstructive pulmonary disease. Conversely, female sex (OR, 1.09 [95% CI, 1.03–1.16]) and a higher burden of symptoms (European Heart Rhythm Association Score III–IV versus I–II: OR, 1.85 [95% CI, 1.74–1.98]) were associated with higher odds of receiving rhythm control at baseline. Among comorbidities, hypertension and coronary artery disease were associated with higher odds of receiving rhythm control, with some evidence (although not statistically significant) for heart failure (Figure 1). When we considered age as a restricted cubic spline in the multivariable logistic regression model, we found that increasing age was nonlinearly and inversely associated with a lower likelihood of receiving rhythm control (Figure S1; *P* for nonlinearity=0.005).

Antithrombotic drugs and other treatments received at baseline according to rhythm control are reported in Figure S2 and Table S1. OAC use was slightly higher in the “rhythm control” group (83.5% versus 81.8%); similar results were observed for non–vitamin K antagonist OAC use (63.2% versus 57.9%). On multivariable logistic regression analysis, rhythm control was associated with higher odds of OAC use at baseline (OR, 1.36 [95% CI, 1.25–1.48]). In patients who received OAC, rhythm control was also associated with higher use of a non–vitamin K antagonist OAC versus vitamin K antagonist (OR, 1.24 [95% CI, 1.15–1.34]).

### Adverse Outcomes According to AF Treatment Strategies

**Figure 2:**
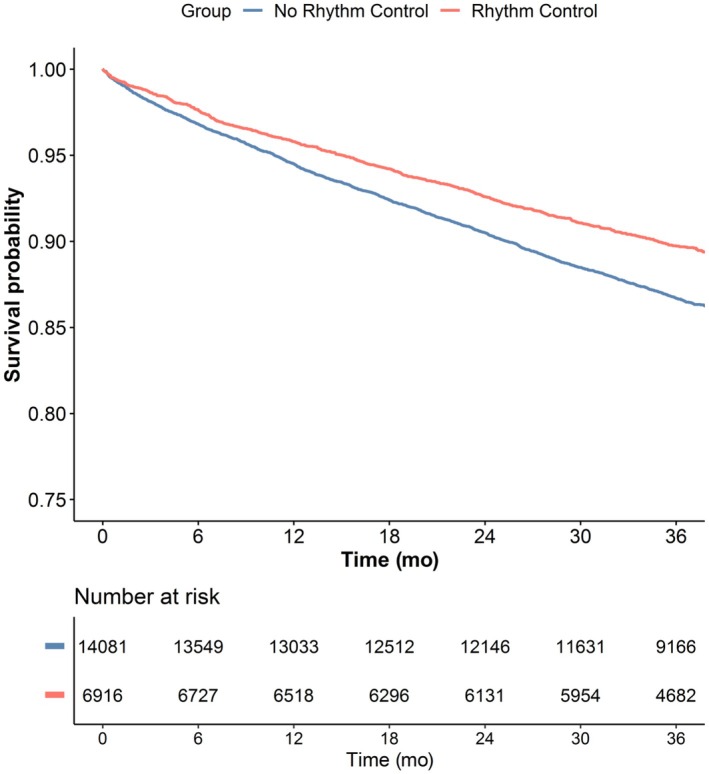
Survival curves for the primary composite outcome of all‐cause death and MACEs, according to rhythm control strategy. Log‐rank 40.37, *P*<0.001. MACE indicates major adverse cardiovascular event.

A total of 20 997 patients were included in the survival analysis (99.7%), with a median follow‐up of 3.0 years (interquartile range, 2.9–3.1). Overall, 2566 primary outcome events occurred during follow‐up. The incidence of the primary outcome was lower in the rhythm control group (Figure 2; *P*<0.001).

Results of the multivariable Cox regression models on the risk of major outcomes according to rhythm control are reported in Table 2. Rhythm control at baseline was associated with lower hazard of the primary composite outcome (HR, 0.88 [95% CI, 0.80–0.96]). Similar results were observed for secondary outcomes, including all‐cause death (HR, 0.88 [95% CI, 0.79–0.98]), thromboembolism (HR, 0.78 [95% CI, 0.66–0.93]) and major bleeding (HR, 0.78 [95% CI, 0.65–0.93]). Some evidence, although without statistical significance, was observed also for MACEs (Table 2). Results of the sensitivity analysis according to the Fine–Gray subdistributional hazard model are reported in Table S3 and were consistent with the main analysis.

**Table 2:** Incidence Rates and Multivariable Cox Regression Models on the Risk of Major Outcomes

	Rhythm control	No rhythm control	Rhythm vs no rhythm control	Rhythm vs no rhythm control
	IR (95% CI) per 100 person‐years	IR (95% CI) per 100 person‐years	Unadjusted HR (95% CI)	Adjusted HR (95% CI)*
Primary outcome				
Composite of all‐cause death, stroke and myocardial infarction	3.6 (3.4–3.9)	4.8 (4.6–5.0)	0.76 (0.69–0.82) *P*<0.001^†^	0.88 (0.80–0.96) *P*=0.006^†^
Secondary outcomes				
All‐cause death	2.7 (2.5–2.9)	3.6 (3.5–3.8)	0.74 (0.67–0.82) *P*<0.001^†^	0.88 (0.79–0.98) *P*=0.016^†^
MACE	2.0 (1.8–2.2)	2.5 (2.3–2.7)	0.80 (0.71–0.90) *P*<0.001^†^	0.89 (0.78–1.01) *P*=0.067
Thromboembolism	1.0 (0.9–1.1)	1.5 (1.4–1.7)	0.65 (0.56–0.77) *P*<0.001^†^	0.78 (0.66–0.93) *P*=0.005^†^
Major bleeding	1.0 (0.8–1.1)	1.4 (1.3–1.5)	0.69 (0.59–0.82) *P*<0.001^†^	0.78 (0.65–0.93) *P*=0.006^†^

HR indicates hazard ratio; IR, incidence rate; and MACE, major adverse cardiovascular event.

* Adjusted for age class, sex, congestive heart failure, arterial hypertension, diabetes, history of stroke/transient ischemic attack, peripheral artery disease, coronary artery disease, type of AF, body mass index categories, history of previous bleeding, chronic obstructive pulmonary disease, symptoms at baseline (according to European Heart Rhythm Association score, III–IV vs I–II), use of oral anticoagulants and geographic region of recruitment.

^†^ *P* value < 0.05.

### Interaction Analyses

**Figure 3:**
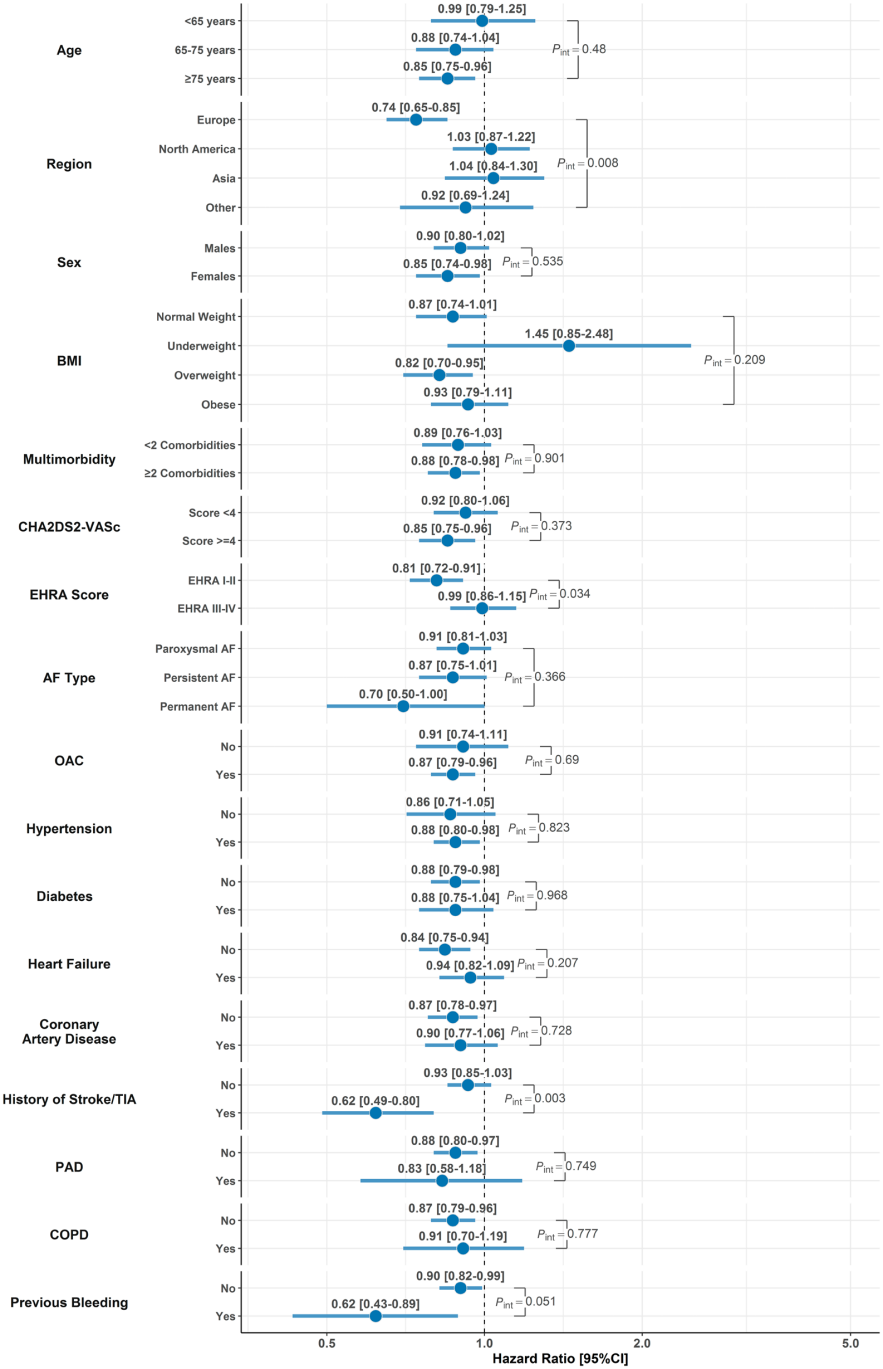
Interaction analyses on the hazard of the primary composite outcome of all‐cause death and MACEs in patients treated with versus without rhythm control, across key subgroups. AF indicates atrial fibrillation; BMI, body mass index; COPD, chronic obstructive pulmonary disease; EHRA, European Heart Rhythm Association; MACE, major adverse cardiovascular event; OAC, oral anticoagulant; PAD, peripheral artery disease; and TIA, transient ischemic attack.

At interaction analysis (Figure 3), we found that rhythm control was associated with larger risk reduction in patients from Europe compared with other regions (*P*_int_=0.008) and in patients with lower baseline European Heart Rhythm Association scores (*P*_int_=0.034). Rhythm control was also associated with greater risk reduction in patients with history of stroke or transient ischemic attack (*P*_int_=0.003). Some evidence, although non–statistically significant was seen in those with prior bleeding (*P*_int_=0.051). No other interaction was observed across subgroups explored (Figure 3).

**Figure 4:**
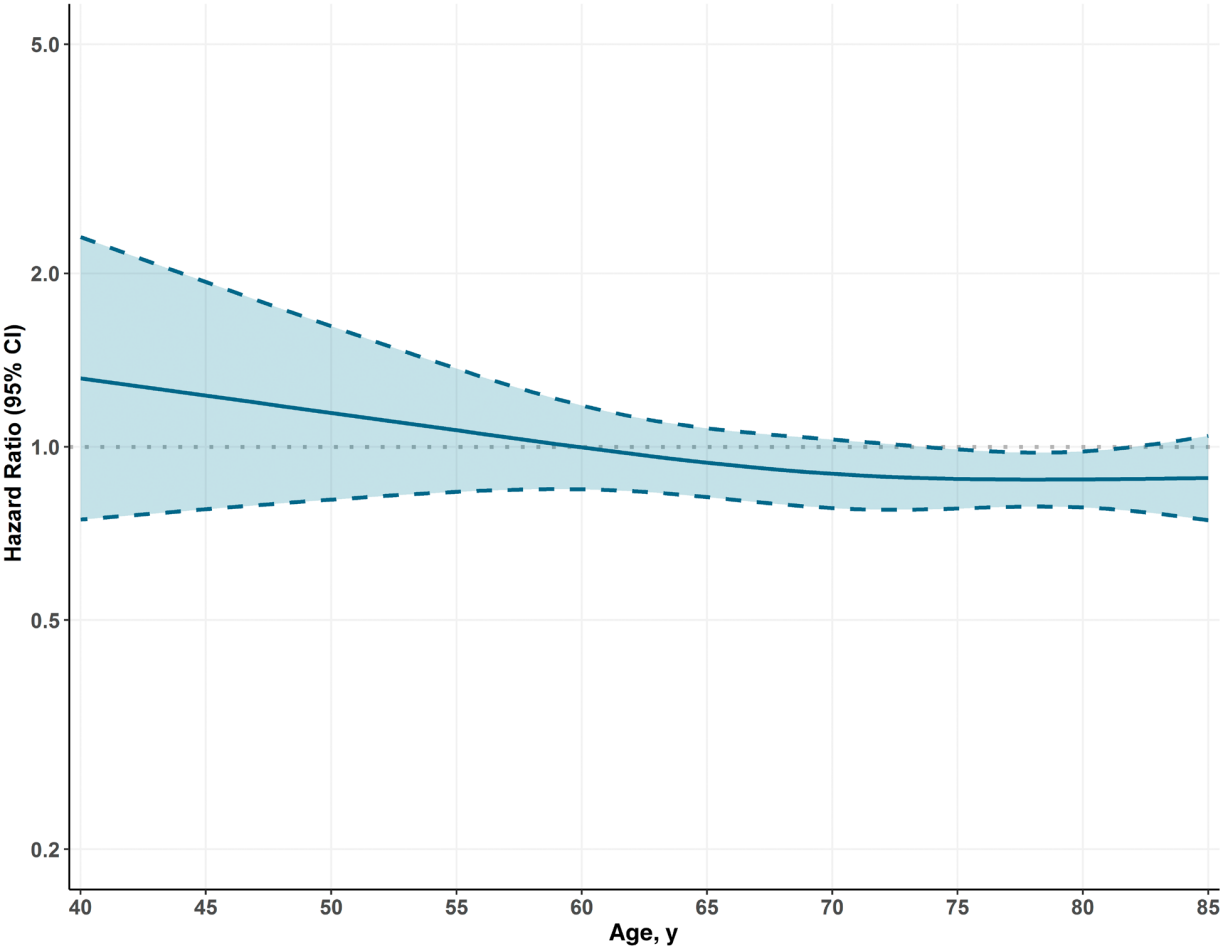
Exploratory analysis on the association of rhythm control with the risk of the primary outcome, across age. *P* for interaction=0.381.

When modeling the interaction of rhythm control with age as a restricted cubic spline, we found no evidence of a significant interaction on the hazard of the primary outcome (*P*_int_=0.381; Figure 4).

## Discussion

On the basis of data from the GLORIA‐AF registry phase III, we found that (1) a rhythm control strategy was implemented in 1 of 3 patients with a recent diagnosis of AF and was associated with younger age, more symptoms, and specific comorbidities (eg, hypertension and coronary artery disease); (2) patients who received rhythm control were also more likely to receive OAC (and more frequently a non–vitamin K antagonist OAC); (3) rhythm control was associated with a lower risk of a composite outcome of all‐cause death and MACEs, as well as with lower risk of other secondary exploratory outcomes; (4) the association with reduced risk of the primary outcome was greater in specific subgroups (eg, those with a history of thromboembolism).

Several historical clinical trials previously demonstrated that rhythm control did not offer benefit over rate control in terms of all‐cause and cardiovascular death.^6^, ^7^, ^8^, ^22^ However, after the EAST‐AFNET 4 trial,^9^ increasing attention has been paid to the implementation of an ERC strategy after the diagnosis of AF. This trend was sustained also by real‐world studies that generally confirmed the benefit of an ERC approach.^12^, ^23^

Our results confirm the effectiveness of a rhythm control strategy in a population of patients with a recent diagnosis of AF and expand the knowledge on the prognostic implications of this approach in a real‐world population of patients with AF. Our findings are broadly consistent with those of the EAST‐AFNET 4 trial, notwithstanding some differences in the 2 cohorts: Beyond the obvious differences in study design, our population was composed of patients with a diagnosis of AF within 3 months of enrollment (4.5 months in Latin America), and all patients had a CHA_2_DS_2_‐VASc score ≥1 (thus slightly different from the inclusion criteria applied in the EAST‐AFNET 4 trial). Finally, we observed a higher proportion of patients treated with amiodarone and a lower use of flecainide compared with the EAST‐AFNET 4 trial, in the context of a similarly low prevalence of AF ablation. Indeed, the higher use of amiodarone was already reported in other cohorts enrolled in similar time periods.^12^, ^24^ Moreover, we also observed significant geographic variation in the use of antiarrhythmics and rhythm control interventions, consistent with previous studies that reported substantial heterogeneity in approaches to AF management, both within and across geographic regions.^25^, ^26^ Of note, the GLORIA‐AF registry included patients with a recent diagnosis of AF, and, as our data suggest, rhythm control was mostly implemented through antiarrhythmic drugs or cardioversion, which are among the first immediate strategies to pursue rhythm control. Indeed, AF ablation may require additional evaluation, time, and availability of experienced centers and referral pathways. Also, at the time of study, there was still limited experience with some ablation techniques. Taken together, all these aspects may have limited the uptake of AF ablation in our study; the increasing use of this approach in more recent years^27^ (also in view of the increasing data on the efficacy and safety of this strategy^28^) will likely translate in higher prevalence of AF ablation in newer cohorts.

We also found that rhythm control was unevenly implemented in real‐world patients with AF, being influenced by the patient's clinical profile. Individuals receiving rhythm control were younger, were more likely women, had less sustained AF, and had more symptoms. Those with cardiovascular comorbidities were also more likely to undergo a rhythm control strategy, as reported in previous observational analyses, with the exception of those with previous thromboembolic events. These results point to a suboptimal implementation of rhythm control in this subgroup of patients at high risk of adverse events. This is notwithstanding previous evidence demonstrating a similar efficacy of ERC in patients with a previous history of stroke,^29^ including a subanalysis of the EAST‐AFNET 4 trial^30^ and the open‐label RAFAS (Risk and Benefits of Urgent Rhythm Control of Atrial Fibrillation in Patients With Acute Stroke) trial.^31^ Our interaction analysis confirms these results, showing how the benefit of a rhythm control approach is even greater in magnitude in patients with a previous thromboembolic events and underlining how such an approach should be carefully considered in these patients.

We also observed a nonlinear, inverse association of rhythm control use with age, reflecting the lower implementation of rhythm control in older people with AF, in line with previous studies.^32^, ^33^ Reasons for these differences may include an expected higher benefit of a rhythm control approach in younger patients (notwithstanding previous reassuring data on its safety and efficacy even at older ages), or in people who are frail.^32^, ^34^ Older adults more frequently present with a higher burden of comorbidities and frailty, and as showed in previous studies, the benefit of ERC is more uncertain and may be mitigated in these patients, compared with younger or nonfrail individuals.^34^, ^35^ These data support a tailored and patient‐centered implementation of ERC, identifying the appropriate strategy according to the patients' clinical risk profile, time to benefit in view of life expectancy, and personal preferences.

We found that rhythm control was associated with higher use of OAC, consistent with a previous analysis of the GLORIA‐AF registry phase II/III that focus on catheter ablation.^36^ Reasons supporting this association include differences in thromboembolic and bleeding risks, and other baseline characteristics, as well as a potentially underlying more intensive and guideline‐adherent treatment of patients who received rhythm control. Indeed, appropriate anticoagulation and symptom‐directed decisions on rhythm versus rate control are both pillars of the holistic or integrated care approach for the management of AF endorsed by international guidelines,^1^, ^2^, ^3^, ^4^ given the association with a markedly reduced risk of major outcomes.^37^, ^38^ Our results suggest that rhythm control and OAC are often offered together, likely in the context of an integrated approach to the treatment of AF.

The association of rhythm control with an overall lower risk of all major outcomes investigated in our analysis have important clinical implications. Particularly, the magnitude of risk reduction that we observed are consistent with those reported in the EAST‐AFNET 4 trial^9^ and previous observational studies.^12^, ^39^ Our data expand knowledge of the effectiveness of rhythm control in several subgroups of patients with a recent diagnosis of AF, including those with cardiovascular comorbidities, consistently with secondary analyses of the EAST‐AFNET 4 trial,^10^, ^30^, ^40^ as well as with other observational studies focusing on specific subgroups of patients with AF, for which a similar benefit of rhythm control approach has been observed.^13^, ^14^, ^41^ Benefits of rhythm control may be even higher in patients with a history of thromboembolism or previous bleeding and may also extend to those with fewer symptoms. We finally observed geographic differences, with a significant interaction pointing toward more benefit of rhythm control in European patients. Such differences need to be confirmed in other studies.

Taken together, our results support a rhythm control approach for patients with a recent diagnosis of AF, in view of the association with improved prognosis; this benefit is consistent across most subgroups. Results from our study confirm the importance of considering rhythm control strategy to manage patients with a recent diagnosis of AF, in accordance with international guidelines,^1^, ^2^ and in the context of a holistic, integrated approach to AF management. Indeed, consideration of a rhythm control versus a sole rate control strategy is a crucial part of the AF care pathway. Choices should be individualized and guided by several factors, including considerations on prognostic benefits, symptoms, and patient's preferences, without applying a “one size fits all” strategy.

### Strengths and Limitations

Our analysis is based on a contemporary, real‐world cohort of patients with a recent diagnosis of AF, thus representing a suitable population to investigate the effectiveness of a rhythm control strategy implemented early after AF diagnosis. The relatively large sample size allowed us to investigate subgroup differences, and to explore factors associated with a rhythm control versus a non–rhythm control strategy in real‐world practice.

Our study also has limitations. First, this is a retrospective analysis; therefore, exposure to a rhythm control versus a non–rhythm control strategy was not randomized, and several factors (including others not included in our analysis) could have influenced the choice of a rhythm control strategy, thus leading to a potential confounding by indication bias in the interpretation of our results. As such, our results should be interpreted with caution, as causal association cannot be demonstrated in retrospective analyses. Additionally, our analysis could not evaluate the relative contribution of the different rhythm control modalities (antiarrhythmic drugs, ablation, cardioversion) on the risk of major outcomes, and further studies are needed to elucidate this. Moreover, we focused on treatment and interventions as collected at baseline by investigators, and we cannot exclude the transitions of some patients to the other strategy during follow‐up; a change in strategy could influence the subsequent risk of outcomes, and this should prompt cautious interpretation of our results. We were not able to consider certain antiarrhythmics drugs that can be used in clinical practice to maintain sinus rhythm in patients with AF (including dofetilide, sotalol, and others). Although our regression analyses were adjusted for several variables (including parameters in the CHA_2_DS_2_‐VASc score), we cannot exclude the contribution of other, unaccounted confounders in the results observed, and particularly on the association of rhythm control with the risk of major outcomes. Moreover, we did not have data on the assessment of atrial cardiomyopathy, which has been described as a major determinant for incidence of AF.^42^ Additionally, no data on the burden of AF were available, nor at baseline or follow‐up; therefore, it was not possible to evaluate effectiveness of rhythm control strategy on this outcome. Finally, our results on secondary outcomes were not adjusted for multiple comparisons and should be therefore interpreted as exploratory and with caution.

## Conclusions

In this large real‐world prospective registry of patients with a recent diagnosis of AF, a rhythm control approach was implemented in one third of patients, being associated with specific clinical characteristics, with a higher use of OACs, and with better outcomes. These findings support the need to consider a rhythm control strategy in patients with recently diagnosed AF, in the context of personalized and integrated management of AF.

## Sources of Funding

This GLORIA‐AF registry was funded by Boehringer Ingelheim GmbH. The authors are solely responsible for the design and conduct of this study, all study analyses, the drafting and editing of the manuscript, and its final contents.

## Disclosures

G.F.R. reports consultancy for Boehringer Ingelheim and an educational grant from Anthos, outside the submitted work. No fees are directly received personally. M.P. is investigator of the AFFIRMO (Atrial Fibrillation Integrated Approach in Frail, Multimorbid, and Polymedicated Older People) project on multimorbidity in AF, which has received funding from the European Union's Horizon 2020 research and innovation program under Grant Agreement Number 899871. G.B. reports small speaker fees from Bayer, Boehringer Ingelheim, Boston, BMS, Daiichi, Sanofi, and Janssen outside the submitted work. He is also the principal investigator of the ARISTOTELES (Applying Artificial Intelligence to Define Clinical Trajectories for personalized Prediction and Early Detection of Comorbidity and Multimorbidity Patterns) project, funded by the European Union within the Horizon 2020 research and innovation program (Grant No. 101080189). B.O. was US co‐coordinator of the GLORIA‐AF registry. M.V.H. has been receiving research grants from the Dutch Healthcare Fund, Dutch Heart Foundation, BMS‐Pfizer, Bayer Healthcare, and Boehringer Ingelheim; and consulting fees from BMS‐Pfizer, Bayer Healthcare, and Boehringer Ingelheim to the institution. G.Y.H.L. has been consultant and speaker for BMS/Pfizer, Boehringer Ingelheim, Anthos, and Daiichi‐Sankyo. No fees are directly received personally. All the disclosures happened outside the submitted work. He is a National Institute for Health and Care Research senior investigator and co–principal investigator of the AFFIRMO project on multimorbidity in AF (Grant Agreement No. 899871), TARGET project on digital twins for personalized management of atrial fibrillation and stroke (Grant Agreement No. 101136244) and ARISTOTELES project on artificial intelligence for management of chronic long‐term conditions (Grant Agreement No. 101080189), which are all funded by the European Union's Horizon Europe Research and Innovation program.

## Supporting information

List of GLORIA‐AF Investigators
Tables S1–S3
Figures S1 and S2
